# Clinical impact of molecular diagnosis in cat allergy

**DOI:** 10.1186/2045-7022-4-S2-P51

**Published:** 2014-03-17

**Authors:** Silvia Uriarte Obando, Joaquín Sastre Domínguez

**Affiliations:** 1 Fundación Jiménez Díaz , Allergy Department, Madrid, Spain

## Background

Allergy to cat is a frequent cause of rhinitis, asthma or contact urticaria. Dander, saliva and urine are sources of cat allergens. The prevalence of sensitization to different cat allergens is not well known.

## Methods

We select 105 sensitized patients to cat. Specific IgE measurement to cat allergens Fel d1, Fel d2 and Fel d4 was performed by ImmunoCAP® and/or microarray ISAC® (ThermoFisher Scientific, Sweden), a value > 0,35 kU/L or >0,3 ISU was considered as positive, respectively. Skin prick test was performed with an ALK-Abelló extract (Denmark). Association of Specific IgE measurements and presence and type of rhinitis or asthma was studied . Statistical analysis was performed using Fisher test and chi-squared test.

## Results

88% of patients had specific IgE to Fel d1, 22% to Fel d2, and 42% to Fel d4. 48% were monosensitized to Fel d1, 7% to Fel d2 and 4% to Fel d4. 86% of sensitized patients to Fel d4 were also sensitized to Fel d1. Fel d2 was associated with severity of rhinitis and asthma (p <0.01, p 0.01, respectively). Fel d4 was associated with presence of asthma symptoms (p<0.04). Direct contact with cats was associated both to persistence and severity of rhinitis (p 0.0003, p 0.0001, respectively). A positive skin prick test to cat was associated with rhinitis and asthma symptoms (p < 0.01, p <0.01, respectively).

## Conclusion

Different patterns of sensitization to cat allergens in patients with cat allergy can help us to predict the severity and persistence of symptoms.

